# Exploring Factors for Prescription and Validation of Actuated Upper Limb Devices: a Cross-sectional Survey of Allied Health Professionals

**DOI:** 10.33137/cpoj.v7i1.43790

**Published:** 2024-09-22

**Authors:** A Galbert, A. Buis

**Affiliations:** Department of Biomedical Engineering, Faculty of Engineering, University of Strathclyde, Glasgow, Scotland.

**Keywords:** Assistive Device, Exoskeletons, Actuated, Powered, Allied Health Professionals, Upper Limb, Design, Outcome Measures, Orthosis, Rehabilitation, Satisfaction, Survey

## Abstract

**BACKGROUND:**

Actuated devices can be beneficial for individuals with upper limb muscle weakness, offering extra force and grip. Utilising this type of assistive device can facilitate daily activities, thereby enhancing independence and overall quality of life. The development of actuated assistive devices has been growing, and current literature shows promise in their clinical use. However, they are not yet medically recommended by global guidelines and councils. Studies have suggested why assistive devices have barriers to access, but actuated devices have not been a focus in these discussions.

**OBJECTIVE(S):**

To address this issue, a survey was conducted among professionals who prescribe and assess upper limb assistive devices. The survey aimed to gather their opinions and quantify the factors that might contribute to the limited use of actuated devices in the field.

**METHODOLOGY:**

A web-based cross-sectional study was designed using Qualtrics, contained 25 items and was conducted between October 2023 and January 2024. The survey was piloted, validated, and ethically approved. Results were statistically analysed, and open questions underwent thematic analysis.

**FINDINGS:**

87 Allied Health Professionals (AHPs) contributed to the survey, with a completion rate of 69% (60/87). Survey respondents predominately worked from the USA (72%). The survey revealed that 66% of respondents felt they did not have sufficient access to assistive devices and 58% indicated that outcome measures could be improved. They also noted that actuated devices needed to better meet user-centric needs. Barriers to prescribing these devices included a lack of awareness, experience and standardised prescription methods. In addition, the limited time with patients made decision-making and validation of an actuated device difficult.

**CONCLUSION:**

AHP's have experience prescribing assistive devices but do not have access, knowledge, or clinical methods to assess the use of actuated devices. Future designs for actuated devices should focus on wearability, comfort, user satisfaction, safety and ease of use.

## INTRODUCTION

Upper limb impairment can affect anyone at any time. Age, injury, disease, and other conditions may present a person with motor impairments such as weakness, paralysis, spasticity, tremors and dystonia. They may have a full recovery to independent life through rehabilitation, but others may require further support. When upper limb motor impairments affect the hands and wrist, which is the focus of this study, activities of daily living (ADLs) are negatively impacted. Functional tasks such as self-care, cooking, and working may be affected which can reduce community engagement and quality of life.^1,2^ In addition, a person may require regular support from caretakers and allied health professionals, these visits and other expenses present financial, resource and time burdens on the medical sector.^3-6^

Assistive devices can be prescribed to support persons who require additional functionality and prehension in their upper limbs. Assistive devices have a history of use in medical and occupational fields.^7,8^ They can range from orthotics to complex machinery such as an exoskeleton. Orthotic devices such as splints and braces are used to stabilise and support the limb which aids in maintaining proper alignment and preventing further injury. Orthotic devices can be custom-made or prefabricated. Exoskeletons are robotic assistive devices which support and enhance movements, they have been used in rehabilitation clinics to aid in restoring motor function. Other assistive devices can include adaptive equipment, arm supports, and mechanical hand and finger exercisers to improve grip through repetitive exercises. The level of support and type of tools should reflect the user's needs. Therefore, an actuated, powered, and active device would be appropriate for users needing additional strength during everyday tasks. These devices provide additional force using motors to attain the power requirements for ADLs. Yet, these devices are not listed as recommended tools in guidelines such as the NICE guidelines.^9^ Current literature on assistive technology in global and low- and middle-income countries has reported barriers such as cost, weight, validation, and poor market conditions.^10,11^ However, actuated and powered devices for the upper limb have not been specifically highlighted in these reports. Due to the recent emergence of actuated assistive devices and the rapid pace of ongoing research, there is a lack of longitudinal studies validating their clinical efficacy.^12^

To explore why actuated assistive devices for hand motor impairments are not recommended, a cross-sectional survey was conducted to gather opinions from stakeholders who assess and prescribe assistive devices. Stakeholder opinion on assistive technology has been conducted previously but focused on service providers,^13^ training needs,^14,15^ and software applications.^16^ In addition, literature that has focused on the design and application of assistive devices for upper limb impairment lacked a stakeholder perspective.^17^ These stakeholders, who support and understand user needs, can provide valuable insights into design factors and outcome measures, aiding in the development and clinical validation of future actuated devices.

A cross-sectional survey is a time-efficient and costeffective method for summarizing the population’s relationship to certain characteristics, behaviors, or outcomes. It is an observational study that collects data from a population (in this case, allied health professionals) at a single point in time to assess the prevalence of various outcomes. For this study, the focus is on gathering the population’s opinions on assistive device design and validation methods. Cross-sectional studies can be conducted through interviews and surveys, a survey was chosen for its efficiency and accessibility.

The study aims to conduct a cross-sectional survey to explore barriers and limitations of actuated assistive devices with a 2-part focus on trends in current assistive devices and recommended outcome measures. The objectives include quantifying important design factors, and investigating trends in assistive devices and outcome measures used for persons with motor impairment in the upper limb. This will contribute to the field of actuated assistive devices and provide a basis for future research in new designs and validation processes to improve prescription.

## METHODOLOGY

### Recruitment

An online cross-sectional survey was designed and conducted from October 2023 to January 2024 in Qualtrics, a web-based software for creating surveys. The research was approved by the University of Strathclyde's Departmental Ethical Committee (DEC.BioMed.2023.348). The participation information sheet and consent forms were integrated into the survey questions (found in the **Appendix**). All collected data was anonymized, and data protection and risk assessment protocols were followed.

Inclusions for participation in the study were limited to those who currently work, or have worked, in the field of upper limb assistive devices, individuals with first or second-hand experience with users requiring assistive devices, who understand English for academic discussion, and those with access to a device capable of completing the online survey. Individuals who have not practised in this field within the last 5 years were excluded.

Four associations in the appropriate fields of the study were contacted for dissemination of the survey. These included the American Society of Hand Therapists (ASHT), the British Association of Hand Therapists (BAHT), the British Association of Prosthetics and Orthotics (BAPO), and the International Society of Prosthetics and Orthotics (ISPO). As part of the approval for dissemination, each association included a validation and piloting process.

ASHT required ethical approval, and the survey was reviewed with feedback from 2 members of the research division. BAHT reviewed the survey with feedback from 2 clinical evidence committee members using the Harvard University Program on Survey Research (PSR) questionnaire.^18^ BAPO required ethical approval and pilottested the survey with 9 members of their research committee. ISPO also required ethical approval. The survey was internally validated using the Checklist for Reporting Results of Internet E-Surveys (CHERRIES) by Eysenbach^19^ and followed Siny Tsang guidelines for developing, translating, and validating a questionnaire.^20^

Once the survey had been approved, the associations disseminated the survey link via posters, newsletters, and mailing lists. We also used social media platforms and conferences to advertise the study. No incentives were provided to fill out the online survey.

### Survey design

The survey was designed in a structured format comprising 25 items which can be found in the **Appendix**: 4 Open questions, 10 Closed questions, 10 Multiple choices, and 1 Likert scale. It starts with the participant information sheet, followed by the consent form. The survey then consisted of 4 main sections focusing on AHPs' perspectives on assistive devices and a second part on the rationale for the lack of prescription.

### Demographics

This section included questions related to participants’ occupations, experience in the field (in years), country of work, and the patient population they interact with. Allied health professionals may have multiple roles, therefore, occupation selection allows for multiple choice.

### Actuated devices

Participants were asked if they prescribed and or assessed assistive devices for hands and wrists, this was required for adaptive questions further on in the survey. They were then asked if they recognised and had experience with the assistive devices listed compiled from current literature^21^ and discussions with clinicians who did not pilot the survey. The list was designed to include a range of devices from static casts to robotic actuated devices. The inclusion of non-actuated devices in the list was important to reduce response bias.

Those who did not prescribe or assess assistive devices had the opportunity to give their perspectives on why they do not. This section also asked for opinions on access to devices and how they are financed.

### Design factors

Design factors were based on a modified Quebec User Evaluation of Satisfaction with Assistive Technology (QUEST 2.0).^22^ Additional modifications included the removal of “access” for “service” items, changes to the ranking labels from “satisfaction” levels to “level of importance”, and item labels were adjusted to better suit actuated devices such as adding mechanical power. Participants would be asked for their opinions and experiences on assistive devices, these open-question responses would undergo thematic analysis to extract additional design factors missed by the QUEST 2.0.

### Outcome measures

This section asked participants if they have experience using outcome measures and let them pick which they had used and recommend from a list made from results of a dataset^21^ and literature.^23^ Respondents were then asked to identify the main limitations to assessing outcomes for patients and if they believed outcome measures were useful and could be improved.

### Validity, Bias, and Limitations

The validity of the survey was assessed by correspondents, ASHT and BAHT committee members, using respected guidelines and questionnaires. These included the PSR questionnaire,^18^ CHERRIES^19^ and subjective judgement from persons in the relevant fields. Due to time constraints, test-retest reliability was not conducted. To reduce the effect of this limitation, time limits were removed, and the questionnaire was designed to take less than 15 minutes.^24^ Estimated time found using the Qualtrics predicted duration programme. The absence of a time limit can aid in higher completion rates as respondents can complete the survey at their own pace.^25^

Coverage bias was expected due to requiring English speakers and an internet connection. In addition, although the survey was disseminated across four different associations via a mailing list, members may have unsubscribed. Coverage bias could not be avoided but was mitigated as best as possible.

To mitigate sampling bias, the target population and sampling frame were clearly defined within the inclusion criteria. Non-response and measurement errors were reduced by circulating the survey, varying question styles, and allowing neutral responses to questions.

### Data analysis

The demographic features of participants were analysed, and participants were grouped by discipline. Interactions between nominal data were summarized to determine significant trends. Multiple-choice questions were analysed using cross-tabulation. These quantitative analyses were performed using Microsoft Excel (version 2408) and Python (3.12) in Visual Studio.

Open-ended questions were coded using thematic analysis following Braun and Clarke's methods.^26^ The process followed an inductive approach in which the data determined the themes. As the open questions were not required, the response rate tended to be lower therefore latent deduction aided in theming the subtext and assumptions underlying the data. Each open question was analysed separately. Themes were then compared across relevant sections to provide qualitative evidence. The thematic analyses were performed by hand on Microsoft Excel.

## RESULTS

### Participants

87 unique responses were collected, with a 69% (60/87) completion rate. All analyses are on the 60 fully completed responses, participant demographic features are present in **Table 1**. Years of experience in the field ranged from 3 months to 52 years (mean=24.42, SD=13.68). AHPs may have multiple job roles, therefore when asking for occupation, participants could choose multiple options leading to 105 total responses from the 60 participants including Occupational Therapists (OT) (44%, 46/105), Hand Therapists (HT) (39%, 41/105), Orthotist (10%, 11/105), Prosthetist (3%, 3/105), Health Care Assistant (HCA) (1%, 1/105), Physiotherapist (1%, 1/105), Rehabilitation Specialist (1%, 1/105), and unspecified other (1%, 1/105).

**Table 1:** List of Participants.

ID No.	Occupation(s)	Work experience (Years)	Country of work	Patient popuiation(s) interacted with	Assessor of assistive device?
**PRESCRIBERS OF ASSISTIVE DEVICES (Rows 1 to 46)**					
**1**	Hand Therapist, Occupational Therapist	13	United States of America	Carpal tunnel syndrome, Epicondylitis, Multiple sclerosis, Parkinson's disease, Peripheral neuropathy, Stroke	Yes
**2**	Orthotist	25	United Kingdom of Great Britain and Northern Ireland	Carpal tunnel syndrome, Cerebral palsy, Duchenne muscular dystrophy, Multiple sclerosis, Parkinson's disease, Peripheral neuropathy, Spinal Cord Injury, Stroke	Yes
**3**	Hand Therapist, Occupational Therapist	38	United States of America	Carpal tunnel syndrome, Epicondylitis, Peripheral neuropathy, Stroke	Yes
**4**	Orthotist	7	United Kingdom of Great Britain and Northern Ireland	Carpal tunnel syndrome, Cerebral palsy, Duchenne muscular dystrophy, Multiple sclerosis, Parkinson's disease, Peripheral neuropathy, Spinal Cord Injury, Stroke	Yes
**5**	Orthotist	4.5	United Kingdom of Great Britain and Northern Ireland	Cerebral palsy, Duchenne muscular dystrophy, Multiple sclerosis, Parkinson's disease, Peripheral neuropathy, Spinal Cord Injury, Stroke, Diabetes^*^, Paediatrics^*^	Yes
**6**	Occupational Therapist	30	United States of America	Carpal tunnel syndrome, Epicondylitis, Stroke	Yes
**7**	Hand Therapist, Occupational Therapist	40	United States of America	Carpal tunnel syndrome, Epicondylitis, Peripheral neuropathy, Stroke, Trauma Injury^*^	Yes
**8**	Hand Therapist, Occupational Therapist	20	United States of America	Carpal tunnel syndrome, Epicondylitis, Peripheral neuropathy	Yes
**9**	Hand Therapist	42	United States of America	Carpal tunnel syndrome, Epicondylitis, Multiple sclerosis, Parkinson's disease, Peripheral neuropathy, Trauma Injury^*^, Amputation^*^, Laceration Repair^*^, General	Yes
**10**	Orthotist	0.25	United Kingdom of Great Britain and Northern Ireland	Cerebral palsy, Multiple sclerosis, Parkinson's disease, Peripheral neuropathy, Spinal Cord Injury, Stroke	Yes
**11**	Hand Therapist, Occupational Therapist	20	United States of America	Carpal tunnel syndrome, Cerebral palsy, Epicondylitis, Multiple sclerosis, Parkinson's disease, Peripheral neuropathy, Stroke	Yes
**12**	Hand Therapist	38	Switzerland	Carpal tunnel syndrome, Epicondylitis, Peripheral neuropathy	Yes
**13**	Hand Therapist, Occupational Therapist	28	United States of America	Carpal tunnel syndrome, Epicondylitis, Trauma Injury^*^, Arthritis, General	Yes
**14**	Hand Therapist, Occupational Therapist, Other (please specify)	52	United States of America	Carpal tunnel syndrome, Epicondylitis, Parkinson's disease, Peripheral neuropathy, Trauma Injury^*^, Laceration Repair^*^	Yes
**15**	Hand Therapist, Occupational Therapist	32	United States of America	Carpal tunnel syndrome, Cerebral palsy, Epicondylitis, Peripheral neuropathy, Stroke	Yes
**16**	Hand Therapist, Occupational Therapist	15	United States of America	Carpal tunnel syndrome, Epicondylitis, Peripheral neuropathy, Stroke, Amputation^*^	Yes
**17**	Hand Therapist, Occupational Therapist	27	United States of America	Carpal tunnel syndrome, Duchenne muscular dystrophy, Epicondylitis, Multiple sclerosis, Peripheral neuropathy, Laceration Repairs^*^, Trauma Injury^*^, General	Yes
**18**	Occupational Therapist	29	United States of America	Carpal tunnel syndrome, Epicondylitis, Trauma Injury^*^	Yes
**19**	Hand Therapist, Occupational Therapist	37	United States of America	Carpal tunnel syndrome, Epicondylitis, Peripheral neuropathy, Stroke	Yes
**20**	Occupational Therapist	39	South Africa	Carpal tunnel syndrome, Cerebral palsy, Duchenne muscular dystrophy, Epicondylitis, Parkinson's disease, Peripheral neuropathy, Spinal Cord Injury, Stroke, Trauma Injury^*^, Laceration Repair^*^, General	Yes
**21**	Occupational Therapist	33	United States of America	Carpal tunnel syndrome, Epicondylitis, Parkinson's disease, Peripheral neuropathy, Spinal Cord Injury, Stroke, Laceration Repairs^*^	Yes
**22**	Hand Therapist, Occupational Therapist	33	United States of America	Carpal tunnel syndrome, Epicondylitis, Peripheral neuropathy, Stroke	Yes
**23**	Hand Therapist, Occupational Therapist	32	United States of America	Carpal tunnel syndrome, Epicondylitis	Yes
**24**	Hand Therapist, Occupational Therapist	28	United States of America	Carpal tunnel syndrome, Epicondylitis, Multiple sclerosis, Parkinson's disease, Peripheral neuropathy, Spinal Cord Injury	Yes
**25**	Occupational Therapist	23	United Kingdom of Great Britain and Northern Ireland	Carpal tunnel syndrome, Cerebral palsy, Epicondylitis, Multiple sclerosis, Parkinson's disease, Peripheral neuropathy, Stroke, Trauma Injury^*^	Yes
**26**	Hand Therapist, Occupational Therapist	50	United States of America	Peripheral neuropathy, Spinal Cord Injury, Burns^*^, Amputation^*^	Yes
**27**	Hand Therapist, Occupational Therapist	33	United States of America	Carpal tunnel syndrome, Cerebral palsy, Duchenne muscular dystrophy, Epicondylitis, Multiple sclerosis, Parkinson's disease, Peripheral neuropathy	Yes
**28**	Hand Therapist, Occupational Therapist	42	United States of America	Carpal tunnel syndrome, Epicondylitis, Peripheral neuropathy, Laceration Repairs^*^, General^*^	Yes
**29**	Occupational Therapist	24	United Kingdom of Great Britain and Northern Ireland	Carpal tunnel syndrome, Epicondylitis, Peripheral neuropathy	Yes
**30**	Hand Therapist, Occupational Therapist	27	United States of America	Carpal tunnel syndrome, Peripheral neuropathy	Yes
**31**	Hand Therapist, Occupational Therapist	9	United States of America	Carpal tunnel syndrome, Duchenne muscular dystrophy, Epicondylitis, Multiple sclerosis, Peripheral neuropathy, Spinal Cord Injury, Stroke	Yes
**32**	Occupational Therapist	22	United Kingdom of Great Britain and Northern Ireland	Carpal tunnel syndrome, Epicondylitis, Peripheral neuropathy	Yes
**33**	Hand Therapist, Occupational Therapist	42	United States of America	Carpal tunnel syndrome, Epicondylitis, Peripheral neuropathy, Orthopedics^*^, Arthritis^*^	Yes
**34**	Hand Therapist, Occupational Therapist	6	United States of America	Carpal tunnel syndrome, Epicondylitis, Peripheral neuropathy, Trauma Injury^*^, Orthopedics^*^	Yes
**35**	Hand Therapist	24	United States of America	Carpal tunnel syndrome, Epicondylitis, Peripheral neuropathy	Yes
**36**	Hand Therapist, Occupational Therapist	40	United States of America	Carpal tunnel syndrome, Epicondylitis, Parkinson's disease, Peripheral neuropathy	Yes
**37**	Hand Therapist, Occupational Therapist	50	United States of America	Spinal Cord Injury, Burns^*^, Amputation^*^, General^*^	Yes
**38**	Hand Therapist, Occupational Therapist	43	United States of America	Carpal tunnel syndrome, Epicondylitis, Peripheral neuropathy, General^*^, Arthritis^*^	Yes
**39**	Orthotist	11	United Kingdom of Great Britain and Northern Ireland	Carpal tunnel syndrome, Cerebral palsy, Duchenne muscular dystrophy, Epicondylitis, Multiple sclerosis, Parkinson's disease, Peripheral neuropathy, Spinal Cord Injury, Stroke	Yes
**40**	Hand Therapist, Health Care Assistant (HCA), Orthotist, Physiotherapist, Prosthetist, Rehabilitation Specialist	3	Nigeria	Cerebral palsy, Stroke	Yes
**41**	Orthotist, Prosthetist	29	Ireland	Carpal tunnel syndrome, Cerebral palsy, Duchenne muscular dystrophy, Epicondylitis, Multiple sclerosis, Parkinson's disease, Peripheral neuropathy, Spinal Cord Injury, Stroke, Amputation^*^	Yes
**42**	Orthotist	10	United Kingdom of Great Britain and Northern Ireland	Carpal tunnel syndrome, Cerebral palsy, Duchenne muscular dystrophy, Epicondylitis, Multiple sclerosis, Parkinson's disease, Peripheral neuropathy, Spinal Cord Injury, Stroke	Yes
**43**	Orthotist	12	United Kingdom of Great Britain and Northern Ireland	Cerebral palsy, Multiple sclerosis, Spinal Cord Injury, Stroke	Yes
**44**	Orthotist, Prosthetist	6	United Kingdom of Great Britain and Northern Ireland	Cerebral palsy, Spinal Cord Injury, Stroke	Yes
**45**	Hand Therapist, Occupational Therapist	6	United States of America	Carpal tunnel syndrome, Epicondylitis, Parkinson's disease, Peripheral neuropathy	No
**46**	Hand Therapist, Occupational Therapist	9	United States of America	Carpal tunnel syndrome, Epicondylitis, Peripheral neuropathy	No
**NON-PRESCRIBERS OF ASSISTIVE DEVICES (ROWS 47 TO 60)**					
**47**	Hand Therapist, Occupational Therapist	26	United States of America	Carpal tunnel syndrome, Epicondylitis, Multiple sclerosis, Parkinson's disease, Peripheral neuropathy, Stroke, Laceration Repair^*^	Yes
**48**	Hand Therapist, Occupational Therapist	32	United States of America	Carpal tunnel syndrome, Cerebral palsy, Epicondylitis, Multiple sclerosis, Parkinson's disease, Peripheral neuropathy, Spinal Cord Injury, Stroke	Yes
**49**	Occupational Therapist	10	United States of America	Carpal tunnel syndrome, Epicondylitis, Orthopedics^*^	Yes
**50**	Hand Therapist, Occupational Therapist	23	United States of America	Carpal tunnel syndrome, Epicondylitis, Parkinson's disease, Peripheral neuropathy	Yes
**51**	Hand Therapist, Occupational Therapist	16	United States of America	Carpal tunnel syndrome, Epicondylitis, Multiple sclerosis, Peripheral neuropathy, Stroke	Yes
**52**	Occupational Therapist	32	United States of America	Carpal tunnel syndrome, Epicondylitis, Peripheral neuropathy, Orthopedics^*^, Laceration Repair^*^	No
**53**	Hand Therapist, Occupational Therapist	13	United States of America	Carpal tunnel syndrome, Epicondylitis, Peripheral neuropathy	No
**54**	Hand Therapist, Occupational Therapist	28	United States of America	Carpal tunnel syndrome, Epicondylitis, General	No
**55**	Hand Therapist, Occupational Therapist	1	United States of America	Carpal tunnel syndrome, Epicondylitis, Parkinson's disease, Peripheral neuropathy, Stroke	No
**56**	Hand Therapist, Occupational Therapist	34	United States of America	Carpal tunnel syndrome, Epicondylitis, Orthopedics^*^	No
**57**	Hand Therapist, Occupational Therapist	37		Carpal tunnel syndrome, Epicondylitis	No
**58**	Hand Therapist, Occupational Therapist	7.5	United States of America	Carpal tunnel syndrome, Osteoarthritis^*^, Trauma Injury^*^, Dupuytren^*^	No
**59**	Hand Therapist, Occupational Therapist	12	United States of America	Carpal tunnel syndrome, Epicondylitis, Peripheral neuropathy, Stroke	No
**60**	Orthotist	10		Duchenne muscular dystrophy	No

Note: ^*^ Patient Populations abstracted from text response

The respondents interacted with a range of patient populations, as presented in **Table 1**. The top prevalent patient conditions included Carpal Tunnel Syndrome (87%, 52/60), Epicondylitis (80%, 48/60), Peripheral Neuropathy (77%, 46/60), Stroke (47%, 28/60) and Parkinson's Disease (37%, 22/60). Muscle weakness tends to be a symptom in these conditions, but pain is also a major factor when deciding on treatment and management of conditions. Current practices may recommend splinting and bracing for the affected upper limb. This approach is well-researched for the patient conditions supported by the respondents.

The respondents primarily worked from the USA (72%, 43/60), followed by the UK (18%, 11/60); Switzerland, South Africa, Nigeria, and Ireland each had 1 survey taker, and 2 participants did not respond to this question. A noticeable USA-centric participation group influenced the results, particularly when respondents were asked about the funding mechanisms for assistive devices. Multiple funding sources can be used within a medical department, so respondents were given the option to select all applicable sources, resulting in 80 responses. Private health insurance was the most prevalent funding source, cited by 43% (34/80) of respondents, followed by self-funded options at 34% (27/80). This distribution reflects the structure of the U.S. medical sector, where a universal healthcare system is not in place.

### Assistive devices

Many of the assistive devices recognised in **Table 2** used electrical stimulation (70%, 42/60); The TENS Stimulator (65%, 39/60) was the most recognised assistive device, and respondents also had the most experience using it (45%, 27/60). In comparison, AHPs did not have experience using powered and actuated devices (15%, 9/60). MyoPro Orthosis was the most recognised and used actuated device. The MyoPro is an American device which uses Electromyography (EMG) to trigger upper limb movement. The discrepancies in **Table 2** may be due to response errors when completing the survey.

**Table 2:** Assistive devices recognised and used by respondents including their key features.

Device Name	Type of device	Device Feature	Count of assistive devices recognised (n)	Count of assistive devices AHPs have working experience using (n)
**TENS Stimulator**	TENS	Passive Electrical Stimulation	39	27
**SaeboMAS**	Anti-gravity support	Clinical Tool	17	8
**None**			13	8
**MyoPro Orthosis**	Powered and actuated device	EMG control	10	5
**EXOTIC exoskeleton**	Powered and actuated device	Rigid exoskeleton	9	1
**ReHand**	Rehabilitation Software	Tablet-based	8	2
**GraspyGlove**	Powered and actuated device	Soft exoskeleton	5	1
**Hand of Hope**	Powered and actuated device	EMG control	5	0
**X-Glove**	Powered and actuated device	Rigid exoskeleton	4	0
**JACO**	Assistive robotic arm	Manual selection	3	2
**SEM Glove**	Powered and actuated device	Soft exoskeleton	3	0
**Tenoexo hand exoskeleton**	Powered and actuated device	Semi-rigid design and EMG control	3	0
**Odstock Microstim**	Neuromuscular electrical stimulation	EMG control	2	2
**TIGER**	Powered and actuated device	Table-based interface and rigid	2	0
**DTSaM Orthosis**	Powered and actuated device	Soft exoskeleton	1	0
**Fesia Grasp Device**	FES	EMG control	1	0
**Handy Rehab**	Powered and actuated device	Rigid exoskeleton	1	2
Benik splint^*^	Customisable orthosis	Soft orthosis	1	1
Dmo Lycra gloves^*^	Customisable orthosis	Soft orthosis	1	1
Ergonomic kitchen tools^*^	Customised tools	Singular function	1	1
Meta grip cmc splint^*^	Customisable orthosis	Rigid orthotics	1	1
**PneuGlove**	Powered and actuated device	Soft exoskeleton	1	0
**SCRIPT Active Orthosis**	Powered and actuated device	Rigid exoskeleton	1	0
**SNU Exo-Glove**	Powered and actuated device	Soft exoskeleton	1	0
**Microstim**	Neuromuscular electrical stimulation	EMG control	0	1
**NESS Handmaster**	Neuromuscular electrical stimulation	Manual selection	0	1

Note ^*^Devices abstracted from text response

13 respondents did not recognize any of the listed devices. However, 62% of these respondents (8/13) indicated that they prescribe assistive devices. This suggests that the curated list of assistive devices did not capture their experiences.

Many AHPs responded negatively (50%, 30/60) or were unsure (17%, 10/60) when asked if they had enough access to assistive devices. Surprisingly, only 6 people felt they “definitely” had enough access to assistive devices.

Nine participants work in workplaces that do not offer assistive devices. All of them are based in the USA (100%, 9/9) and support patients with carpal tunnel syndrome (100%, 9/9) and epicondylitis (100%, 9/9). The majority are occupational therapists (89%, 8/9). Reasons why assistive devices are not offered by this group were extracted from their open-ended responses and analyzed using thematic analysis. From the nine responses, five themes emerged: (1) AHPs’ belief that the patient population is not appropriate for devices, (2) the patient population is not large enough, (3) AHPs lack exposure to assistive devices, (4) a lack of availability of devices, and (5) the cost of the devices.

“Not enough clients coming that need them [assistive devices] on a regular basis. If we had a sporadic client needing one, we would research and try to reach out for options”

(OT, USA, 32) (Occupation, country, experience in years)

Although this group of respondents did not routinely offer assistive devices in their workplace, the reasons mentioned above overlap with the thematic analysis of the opinions and experiences other AHPs had with assistive devices shown in **Table 3**.

**Table 3:** Main theme and subthemes: opinions and experiences of 13 AHPs on assistive devices for hands and wrists.

Main theme	Sub-theme	Mentions (n)	Defining Statement (occupation, country, experience in years)
**Device Design**	Function	5	“Multifunctional use, patients won't use it if it helps with only 1 [activity]” (HT/OT, 33, USA) ^*^
	Comfort	2	“In my experience, if an AD is not extremely comfortable and easy to use, they usually end up not being used.” (HT/OT, 6, USA)
	Durability	2	“Ability of the patient to obtain a replacement or extra items” (HT/OT, 50, USA)
	Weight	1	“If they do not have proximal strength to be able to lift and manipulate the device, what good is it?” (HT/OT, 32, USA)
**Awareness**	Lack of experience	3	“Very limited experience unfortunately -1 could have used more information/experience to treat patients” (HT/OT, 1, USA)
	Lack of knowledge	3	“I am not familiar with the list of adaptive equipment in your international list.” (HT/OT, 43, USA)
**Prescription**	Unclear methods	5	“Need for clearly defined way to assess if patient is appropriate for the assistive device” (HT/OT, 40, USA)
**User**	Adaption	4	“Patients are very quick to adapt their movements after an injury, and if they can use the opposite hand, they figure out how to quickly without the need of adapted equipment to assist.” (HT/OT, 6, USA)
**Cost**	Cost efficient alternatives	2	“Many times just putting a wrist and hand in a more functional position through static custom splint fabrication can be a low-cost and effective way to address many ADL goals.” (HT/OT, 32, USA)

* (Occupation, country, and experience in years)

**Figure 1:**
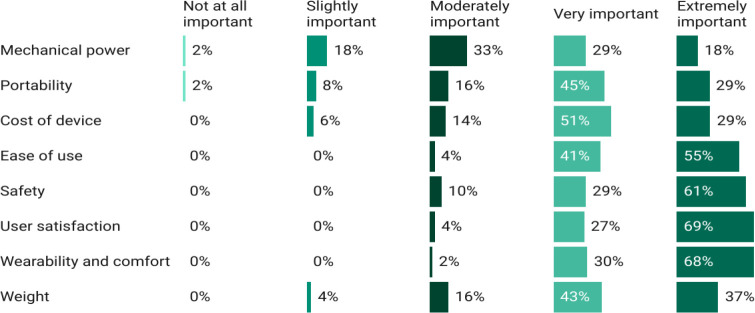
Importance of design features based on Likert scale responses.

Both thematic analysis (**Table 3**) and the Likert responses (**Figure 1**) showed similar factors which affect the prescription of actuated devices. AHPs had concerns about devices’ designs not being multifunctional and uncomfortable for users. User-centric factors (as opposed to mechanical factors) such as comfort, satisfaction, safety and ease of use have a strong level of importance across all demographics. Yet if the device is too heavy or not versatile enough, the users may adapt to not needing one. When comparing the devices used (**Table 2**) with the factors shown in **Figure 1**, the right balance of these factors is difficult to determine. For instance, weight is considered less important than comfort but ensuring a device’s weight and weight distribution is minimalized is often a physical attribute to determine comfort.

### Outcome measures

**Figure 1** quantifies well-established factors of importance pertaining to design, while **Table 3** captures themes often forgotten. In **Table 3**, AHPs felt that there were unclear prescription methods and a lack of awareness of these devices. To better understand prescription methods, the latter half of the survey focused on outcome measures. When and which types of outcome measures are used are essential for tracking improvements in users’ functionality and validating assistive devices. 71% (43/60) of AHPs had experience using outcome measures for upper limb assessment, while 12% (7/60) had some experience and 17% (10/60) had no experience. Most respondents (72%, 43/60) found outcome measurement tools useful while the remainder were unsure (17/60).

Although the majority found them useful, 58% (35/60) believed outcome measurement tools could be improved.

Outcome measures may be used for various reasons. Therefore, AHPs were asked to select all applicable options for when they use outcome measures. Results showed that they are used to assess patients’ needs (44%, 48/108), and to evaluate patients during (20%, 22/108) and after (19%, 20/108) the use of assistive devices. They were often not used to evaluate the assistive device (17%, 18/108). Survey responses from AHPs regarding upper limb outcome measures and tools revealed that many of the measures they had used were not recommended, as shown in **Table 4**. The variety of outcome measures recommended span from simple observational tasks such as the 9HPT to patient-reported outcomes. AHPs often recommended an arsenal of outcome measures tests and rarely relied on a singular test to capture hand/wrist assessment.

**Table 4:** Outcome measures and tools AHPs have experience using and which they recommend, alongside discrepancies between what is recommended and what has been experienced.

Outcome measures and tools	Outcome measures AHPs have experience with (n)	Outcome measures AHPs recommend using for hand/wrist assessment (n)	Difference in recommended tools against experienced
Range of Motion tests	49	43	−6
Pain (self-reported)	45	41	−4
Disabilities of the Arm Shoulder and Hand Questionnaire (DASH)	43	38	−5
Nine Hole Peg Test (9HPT)	42	30	−12
Purdue Pegboard test (PPT)	36	15	−21
Jebsen-Taylor Hand Function tests (JTHFT)	28	12	−16
Other (please specify)	17	13	−4
Ashworth scale	12	6	−6
Box and Blocks Test (BBT)	11	7	−4
Motor Assessment Scale (MAS)	5	3	−2
Southampton Hand Assessment Procedure (SHAP)	5	2	−3
Force Control tests	3	5	2
Patient-Rated Wrist Evaluation (PRWE)^*^	3	2	−1
The Minnesota manual dexterity test (MRMT)^*^	3	0	−3
Upper Extremity Functional Index^*^	3	2	−1
Fugl-Meyer Assessment (FMA)	2	1	−1
Functional Dexterity Test^*^	2	2	0
Patient rate wrist/hand evaluation (PRWHE)^*^	2	2	0
Patient-Specific Functional Scale (PSFS)^*^	2	3	1
Quick DASH^*^	2	0	−2
Action Research Arm Test (ARAT)	1	0	−1
Corbett Targeted Coin Test^*^	1	1	0
Graded Redefined Assessment of Strength, Sensibility and Prehension (GRASSP)	1	0	−1
Grooved peg test^*^	1	1	0
Mankoski Pain Scale^*^	1	0	−1
modified Moberg^*^	1	0	−1
Neck and Upper Limb Index (NULI)^*^	1	0	−1
pinch/grasp strength^*^	1	1	0
Shoulder Pain and Disability Index (SPADI)^*^	1	0	−1
Sollermans^*^	1	0	−1
The Michigan Hand Outcomes Questionnaire (MHQ)	1	1	0
Thumb Disability Examination (TDX)^*^	1	0	−1
Timed functional tasks^*^	1	1	0
Wolf Motor Function Test (WMFT)	1	0	−1
Depends on condition^*^	0	1	1

Note: ^*^ Outcome measures abstracted from text response

Alongside knowing which outcome measures are recommended, participants expressed the limitations of outcome measures. Time constraints are often a limitation in the medical sector, and immense pressure is noticed on healthcare providers^27^ which is reflected in **Table 5**. The results of the thematic analysis, presented in **Table 6**, highlight poor functionality of outcome measures as a recurring theme. Improvements were identified and included using handedness, using a bank of ADLs, including don and doff features, satisfaction levels, and objective tasks as part of the outcome measure. Some of the responses in the functionality theme overlap with relevance and documentation. Respondents wanted outcome measures to be a method of seeing patients improve, yet choosing which outcome measures to use was an issue.

**Table 5:** Main factors limiting AHPs from assessing outcomes for patients requiring hand and wrist assistive devices.

Limiting factors	Mentions (n)	Respondents affected by limiting factor (%)
Time with patients	37	63
Lack of equipment	27	45
Lack of skills or training	21	35
None^*^	3	5
Cost^*^	2	3
Hygiene protocols^*^	1	2
Inappropriate use^*^	1	2

Note: ^*^ Factors abstracted from text response

**Table 6:** Main themes: opinions and experiences of 29 AHPs on outcome measures for hands and wrists.

Theme	Defining Statement (occupation, country, experience in years)	Mentions (n)
**Functionality**	“Basic self-reported outcome measures like QuickDASH don't distinguish one hand vs two for the activities.” (HT, USA, 42)^*^	20
**Relevance**	“outcome measures are important to demonstrate that what we are doing in therapy is being effective. 1 have yet to find one that is really good. We use the Quick DASH because our physicians use it” (HT/OT, USA, 40)	15
**Documentation**	“We need a standardised assessment to help compare between patients but also document a patient's own journey” (Orthotist, UK, 12)	9
**Time**	“if we are seeing them for a one-time visit (more and more common in the US), then where is the time for an outcome study?” (HT/OT, USA, 32)	6
**Inclusiveness**	“Every patient is unique in their injury and their needs, and outcome measurements should better accommodate for this.” (HT/OT, USA, 6)	5
**Financing**	“useful if funding source understands the assessment. I usually document the rational of the measure for the funding source and try to compare the patient's abilities with individuals without the problem.” (HT/OT, USA, 50)	4

* (Occupation, country, and experience in years)

The involvement of funding sources affected the use of outcome measures for three AHPs in the USA. Some funding sources dictate which outcome measures to use, while others allow AHPs to choose as quoted in the financing theme. When these AHPs were told which outcome measures to use by a funding source, such as an insurance company, there were negative responses as quoted below:

“Often outcome measurements are dictated by the company you work for or the insurance plan - typically these are not the most effective measurement tools that we have available”

(HT/OT, USA, 23) (Occupation, country, experience in years)

## DISCUSSION

### Principal findings

The study aimed to explore barriers and limitations of prescribing actuated assistive devices using a cross-sectional survey of allied health professionals. The results indicate that respondents did not recognise nor have experience using state-of-the-art actuated assistive devices, they also had concerns about the design of the devices and methods of prescription. The survey investigated validation methods used by AHPs and concerns about the functionality and relevance were prevalent. AHPs lacked time with patients, equipment and training to conduct outcome measures for using assistive devices, furthering the barriers for prescribing these devices.

87 responses were collected, with 60 complete responses. These respondents were AHPs across the globe from the USA, the UK, Switzerland, South Africa, Nigeria, and Ireland. They supported patients with a multitude of conditions, yet these patients were not all suitable for an upper limb actuated device based on current treatment methods.

Common features of the assistive devices recognised and recommended use a form of electrical stimulation and are distributed in the American marketplace. We can conclude that the US-centric perspective (72% of respondents, 43/60) influenced results, this was noticed in how devices are financed, the reduced importance of the cost of devices, and how financing influences outcome measures. The cost of the devices was considered a less important factor when prescribing an assistive device likely due to the purchasing method of devices.

TENS and EMG devices were expected to be well recognised as they have a long history of use^28^ compared to the state-of-the-art nature of actuated assistive devices.^29^ The wealth of evidence to support TENS and the large selection of devices easily available for purchase makes it an accessible device for self-funded US citizens. In addition to self-funded citizens, privately funded services would prioritize FDA-approved medical devices^30^ which may reduce the stock of international market options for assistive devices, in turn reducing accessibility.

The responses in **Table 3** address the importance of awareness of new technology, the design of the device, how to prescribe devices and user’s adaption to not requiring a device. As muscle weakness and pain affect people differently, devices must be chosen to best suit the needs of the user. An actuated device may fulfil user’s requirements, but decision-making methods for selecting actuated devices are not readily available. Frameworks for selecting assistive technology devices will likely be modified overtime to incorporate actuated devices,^31^ and the mechanical functionality of these devices varies vastly which makes being aware of all the different styles very difficult. Some AHPs were unfamiliar with the actuated devices listed in **Table 2** and suggested they would research on a case-by-case basis for a device, if it would seem useful for a patient. The process of researching and prescribing a device uniquely for a patient is an appropriate method, but time with patients is a considerable barrier (**Table 5** and **Table 6**). One-time visits are a considerable limitation for supporting potential assistive device users, especially if aiming to use outcome measures as quoted in **Table 6**. This poses an additional constraint to documenting a patient’s improvements and any comparison of their functionality with a baseline. As time is an obstacle for both assessing a user's functionality and researching which device would be useful, it is important to understand how to reduce time spent on these tasks.

Outcome measures were used to assess patient’s needs, yet thematic analysis (**Table 6**) showed that the outcome measures used in the workplace did not capture the patient’s functionality. There is an immense list of outcome measures available, but decision-making varied in the demographic groups. Some AHPs chose relevant validation methods whereas some were decided for them by funding sources. Therefore, depending on how much time the AHP had with a patient, finding a relevant outcome measure that checks user functionality is a barrier. In addition, the patient may only have a one-time visit therefore the process of assessing how well the assistive device functions for the user does not get recorded and limits clinical evidence available for their use.

To reduce these limitations, 3 suggestions could be investigated for future research: 1) develop a decision-making tool to help AHPs select appropriate outcome measures based on the available time and equipment; 2) adapt existing, well-established outcome measures to enhance their relevance; or 3) provide patients with a quantitative, longitudinal outcome measure tool to track their functionality and experiences with the actuated device.

The limitations in using outcome measures focused on lack of time, equipment and training. These barriers are reflected in literature in the fields of physical therapy,^32^ hand therapy^33^ and AHPs.^34^ To see if this was reflected in the outcome measures, **Table 7** shows that the more commonly used outcome measures do not take a considerable time to complete (average 14 minutes), nor require complex equipment. However, **Table 7** assumes the outcome measurement tools are set up and only one test is conducted per visit which is unrealistic. The potential contrasting views on the limitations of outcome measures (**Table 5** and **Table 6**) and the ones used in the workplace (**Table 7**) were not questioned as part of the survey, therefore, it is hard to distinguish the source of the contrast. But a reoccurring theme for those who used outcome measures was that when time, equipment and training were not a limitation, the outcome measurement tools chosen were still often irrelevant and did not adequately assess users' functionality.

**Table 7:** A breakdown of the top ten most experienced outcome measures shown in Table 4: equipment, cost, time and skill required to complete.

Outcome measures and tools	Equipment	Cost (£, $)	Time (minutes)	Skill level^*^	References
Range of Motion tests	Goniometer and inclinometer	£15, $5-$100	10	Medium	35.36
Pain (self-reported)	Paper or screen	0	10	low	37.38
DASH	Paper or screen	0	5-10	low	39-41
9HPT	9-hole peg test kit and stopwatch	£9.99-£75, $84	5	low	42 43
PPT	Purdue pegboard and stopwatch	£200, $150	5-10	low	44 45
JTHFT	Test kit (stopwatch, chair, table, paper, clipboard, cards, coffee can, paperclips, beans, spoon, board, clamp, red wooden checkers, cans)	£335, $300-$500	30	Medium	46 47
Ashworth scale	Paper or screen	0	15	Medium	48
BBT	Wooden box, wooden cubes, partition stopwatch	£250,$200	5-10	low	49 50
MAS	Test kit (stopwatch, jellybeans, cup, rubber ball, stool, comb, spoon, pen, teacups, water, jar, table)	Estimated £58.8, $77	15	Medium	5152
SHAP	Test kit (backboard, door handle and zip, shape form-board, foam insert, timer unit, lightweight abstract objects, heavyweight abstract objects, lock and key, zip, coins, buttons, plasticine block, knife, notecard, glass jar with lid, glass jug, cardboard juice carton, empty tin with plastic lid, metal arrow unit, screwdriver)	£2150, $2833	20	Medium	53

Note: ^*^Skill level is assumed low if data collected is observational

### Limitations

This cross-sectional study has several limitations to consider. Due to the methodology, self-reported data and convenience sampling may lead to overgeneralization of results. The sample was not fully representative of all upper limb assistive device prescribers and assessors. However, this limitation highlights the barriers to prescribing and assessing these devices faced by our participants, which warrants further investigation with more stakeholders.

Despite international dissemination, there was a high percentage of American participants, limiting comparisons with other countries. This was partly due to the larger size of the ASHT mailing list (7,000+ members) compared to other associations (<3,000 members). The US-centric perspective did reveal how financing influences the assistive technology marketplace and outcome measure decisions. This underscores the need for future research to include surveys of non-AHP stakeholders, such as users, manufacturers, and policymakers.

The survey questions, which incorporated previous literature for curating lists and validated methods like QUEST 2.0 for design factors of importance, may have lacked flexibility and potentially influenced respondents. However, text fields were provided for additional responses, and results showed that these factors did not significantly influence responses, particularly regarding actuated devices which had low levels of recognisability.

Despite these limitations, our study identifies factors influencing the prescription and validation of actuated upper limb devices. Future research could address improving the stakeholder representation and should tailor the methodology and questions to be more inclusive of factors missed within this study.

## CONCLUSION

A cross-sectional survey quantifying AHPs perspectives on assistive devices and outcome measures was conducted. We identified important factors for prescribing an actuated device. These factors include design requirements, awareness of devices and decision-making support. The population who responded to our survey found many assistive devices to be uncomfortable for users, too heavy, not versatile enough and that users would adapt to not needing one. For an actuated device the weight, cost of the device, mechanical power and portability were considered not as important as other design features. Results showed this may be due to the respondents’ lack of exposure to actuated assistive devices and unclear methods to prescribe a relevant device. In addition, for this group of AHPs, their patient population may not find an actuated device functional or relevant. Our study also shows that outcome measures were rarely used to assess assistive devices, which means their clinical evidence will not increase to improve market exposure.

Conducting outcome measures for patients faced many limitations. AHPs found these tools useful, but due to time constraints, and lack of equipment and training, they were not used regularly. Respondents wanted to use these tools to track the user's functionality, but patients may only attend a one-time visit. To better support AHPs, decision-making tools, training and modifying outcome measures would be appropriate. Increasing the market presence of actuated devices would also increase stakeholder engagement. In the future, researchers should use validation methods that tackle wearability, comfort, user satisfaction, safety and ease of use of their device. These validation methods should encompass observational outcomes used in clinical settings and users' perspectives.

## DECLARATION OF CONFLICTING INTERESTS

The authors declare no conflict of interest.

## AUTHORS CONTRIBUTION

**Angel Galbert:** Study conception and design, data collection, analysis and interpretation of results, draft manuscript preparation, and manuscript revision.

**Arjan Buis:** Supervision, study conception and design, and manuscript revision

All authors have read and approved the final submitted manuscript.

## SOURCES OF SUPPORT

ESPRC doctoral training grant (EP/S02249X/)

## APPENDIX

### Survey Flow

- PERMISSIONS (2 Questions)
- DEMOGRAPHICS (5 Questions)
- ASSISTIVE DEVICES (10 Questions)
- OUTCOME MEASURES (8 Questions)

#### PERMISSIONS

##### Q1: Participant Information Sheet

**Name of department:** Biomedical Engineering

**Title of the study:** Analysis of current hand and wrist assistive devices, and requirements for a future device

**Introduction**

My name is Angel Galbert, and I am a PhD student within the department of Biomedical Engineering at the University of Strathclyde, Glasgow, United Kingdom. I am undertaking this study as part of my research in hand and wrist assistive devices. I am conducting XXXXX research under the supervision of Chief Investigator – Arjan Buis, department of Biomedical Engineering.

**What is the purpose of this research?**

According to the National Institute for Health and Care Excellence (NICE), the population of those affected by muscle weakness in the hands and wrist continues to grow. Conditions such as stroke, spinal cord injury paralysis, Parkinson’s disease and cerebral palsy have shown to affect the hand and wrist ability to function in activities of daily living and therefore reduce quality of life. Assistive devices are being incorporated in the retraining and recovery for some of these conditions, but rehabilitation treatment and assistance may be required outside of a clinical setting. Assistive devices may also provide additional strength, function and prehension to users wanting independence and support.

Previous studies have designed assistive devices that can be used in everyday life in the form of exoskeletons and powered orthoses. These devices show mechanical potential but lack clinical outcome measures when testing. The lack of clinical outcome measures makes it difficult to assess usability. It also makes assistive devices difficult to compare for decision making when prescribing.

This research aims to determine the requirements for an assistive device for use in everyday living by gathering opinions on current devices, user needs and outcome measures. This research will provide the foundations for designing a device with a user-centred approach.

**Do you have to take part?**

No. You do not have to take part as participation is voluntary. If you do not wish to take part in the study, there is no consequences of doing so. You have the right to withdraw from the research without detriment.

**What will you do in the project?**

You will begin by following a link to the survey. The consent form is on the first page of the survey. Please answer the questions on the consent form to confirm that you understand. If you give consent to take part in the study, you will click the button to continue and will be directed on to the survey questions. If you decline to give consent, you will be able to navigate out of the survey. The survey includes questions about your background, experience with hand and wrist assistive devices and upper limb outcome measures. You will also be invited to share your general thoughts on hand and wrist assistive devices. The survey should take no longer than 15 minutes to complete. The screen will state “Thank you for completing the survey” to mark the end of the survey and confirm that your responses have been saved. The survey will be live until December 4th, 2023, and you will be able to take the survey at any point within this period. No monetary incentive will be provided for taking part in the survey. As the survey is online, there are no expenses associated with travel costs.

**Who should take part?**

We are looking for participants who work or have worked with persons with muscle weakness in the hands and wrist. You should have experience of this work within the past 5 years. This may include, but is not limited to, hand therapists, orthotists, prosthetists, occupational therapists, physiotherapists, personal trainer, rehab specialist, assistive technology consultant, medical sales representative, engineers, nurses, doctors, or surgeons. Participants must be able to read and comprehend English.

**What information is being collected in the project?**

Information collected includes your experience with hand and/or wrist assistive devices, your experience with upper limb outcome measures, and opinion on requirements for an assistive device. None of the information collected will be identifiable. Who will have access to the information? The study is conducted in lines with the University General Data Protection Regulation (GDPR) Policy. The University Privacy Notice can be found online on the following website: https://www.strath.ac.uk/research/researchknowledgeexchangeservices/universityethicscommittee/informationsheetconsentform/

Information that you provide will remain confidential. All data will be stored on a secure platform accessed only by the investigators. Only non-identifiable information will be used when findings from the study are shared.

Where will the information be stored and how long will it be kept for?

Data obtained from this study will be stored on the University’s secure platform and accessed only by named researchers. Access and destruction of the data will be according to the University of Strathclyde Data Protection Policy and GDPR. As the data is anonymous, the consent form and anonymous data may be kept indefinitely.

**What happens next?**

Thank you for your attention and time reading the Participant Information.

If you would like to find out more about the project or wish to ask questions before participation, please contact the researchers.

Your responses to the survey will be anonymous. The results of this study will be reported in the researcher’s (Angel Galbert) PhD thesis and may be published in other academic papers, presentations and/or conferences. As the survey is anonymous, the identities of the participants are unknown to investigators and will therefore not be published.

**Researcher contact details:**

Angel Galbert, PhD student

Department of Biomedical Engineering, University of Strathclyde.

Email: Angel.Galbert@Strath.ac.uk

**Chief Investigator details:**

Arjan Buis, Reader

Department of Biomedical Engineering, University of Strathclyde.

Email: Arjan.Buis@Strath.ac.uk

This research was granted ethical approval by the Department of Biomedical Engineering Ethics Committee, University of Strathclyde.

If you have any questions/concerns, during or after the research, or wish to contact an independent person to whom any questions may be directed or further information may be sought from, please contact:

Linda Gilmour

Secretary to the Departmental Ethics Committee, Department of Biomedical Engineering, University of Strathclyde.

Tel: 0141 548 3298

**Email:** Linda.Gilmour@Strath.ac.uk

- **I confirm I have read the participant information sheet (1)**

##### Q2: Consent Form

**Please read of the following statements and check the box below to confirm:**

- **I confirm** that I have read and understood the Participant Information Sheet for the above project and the researcher has answered any queries to my satisfaction.
- **I confirm** that I have read and understood the Privacy Notice for Participants in Research Projects (https://www.strath.ac.uk/research/researchknowledgeexchangeservices/universityethicscommittee/informationsheetconsentform/) and understand how my personal information will be used and what will happen to it (i.e. how it will be stored and for how long).
- **I understand** that my participation is voluntary and that I am free to withdraw from the project at any time, up to the point of completion, without having to give a reason and without any consequences.
- **I understand** that anonymised data (i.e. data that do not identify me personally) cannot be withdrawn once they have been included in the study.
- **I understand** that any information recorded in the research will remain confidential and no information that identifies me will be made publicly available.
  - Yes. I consent to being a participant in the project. (1)
  - No. I do not consent to being a participant in the project. (2)

***Skip To: End of Survey If Q2 = No. I do not consent to being a participant in the project.***

#### DEMOGRAPHICS

##### Q3: What is your occupation?

- Assistive Technology Consultant (1)
- Hand Therapist (2)
- Health Care Assistant (HCA) (3)
- Medical Doctor (4)
- Nurse Specialist (5)
- Occupational Therapist (6)
- Orthotist (7)
- Physiotherapist (8)
- Prosthetist (9)
- Rehabilitation Specialist (10)
- Other (please specify) (11)

##### Q4: Have you worked in this field within the last 5 years?

- Yes (1)
- No (2)

***Skip To: End of Survey If Q4 = No***

##### Q7: Which patient population(s) do you interact with?

- Carpal tunnel syndrome (1)
- Cerebral palsy (2)
- Duchenne muscular dystrophy (3)
- Epicondylitis (4)
- Multiple sclerosis (5)
- Parkinson's disease (6)
- Peripheral neuropathy (7)
- Sarcopenia (8)
- Spinal Cord Injury (9)
- Stroke (10)
- Other (please specify) (11) __________________________________________________

#### ASSISTIVE DEVICES

##### Q8: Do you prescribe assistive devices for hands and wrists?

- Yes (1)
- No (2)

##### Q9: Do you assess assistive devices for hands and wrists?

- Yes (1)
- No (2)

##### Q10: Which assistive device(s) have you heard of?

- ArmMotus (1)
- DTSaM Orthosis (2)
- EXOTIC exoskeleton (3)
- Fesia Grasp Device (4)
- Gloreha Lite glove (5)
- GraspyGlove (6)
- Hand of Hope (7)
- Handy Rehab (8)
- INTFES (9)
- JACO (10)
- MAHI EXO (11)
- MeCFES (12)
- Microstim (13)
- MyoPro Orthosis (14)
- NESS Handmaster (15)
- Odstock (16)
- PneuGlove (17)
- ReHand (18)
- ReIn-Hand system (19)
- RUPERT (20)
- SaeboMAS (21)
- SCRIPT Active Orthosis (22)
- SEM Glove (23)
- SNU Exo-Glove (24)
- Tenoexo hand exoskeleton (25)
- TENS Stimulator (26)
- TIGER (27)
- X-Glove (28)
- None (29)
- Other (please specify) (30) ______________________________________________

***Display This Question: If Q8 = Yes***

##### Q11: Which assistive device(s) have you worked with?

- ArmMotus (1)
- DTSaM Orthosis (2)
- EXOTIC exoskeleton (3)
- Fesia Grasp Device (4)
- Gloreha Lite glove (5)
- GraspyGlove (6)
- Hand of Hope (7)
- Handy Rehab (8)
- INTFES (9)
- JACO (10)
- MAHI EXO (11)
- MeCFES (12)
- Microstim (13)
- MyoPro Orthosis (14)
- NESS Handmaster (15)
- Odstock (16)
- PneuGlove (17)
- ReHand (18)
- ReIn-Hand system (19)
- RUPERT (20)
- SaeboMAS (21)
- SCRIPT Active Orthosis (22)
- SEM Glove (23)
- SNU Exo-Glove (24)
- Tenoexo hand exoskeleton (25)
- TENS Stimulator (26)
- TIGER (27)
- X-Glove (28)
- Other (please specify) (29) __________________________________________________

***Display This Question: If Q8 = No***

##### Q12: Do you offer assistive devices for hands and wrists in your workplace?

- Yes (1)
- No (2)

***Display This Question: If Q12 = Yes***

##### Q13: Which assistive device(s) are offered in your workplace?

- ArmMotus (1)
- DTSaM Orthosis (2)
- EXOTIC exoskeleton (3)
- Fesia Grasp Device (4)
- Gloreha Lite glove (5)
- GraspyGlove (6)
- Hand of Hope (7)
- Handy Rehab (8)
- INTFES (9)
- JACO (10)
- MAHI EXO (11)
- MeCFES (12)
- Microstim (13)
- MyoPro Orthosis (14)
- NESS Handmaster (15)
- Odstock (16)
- PneuGlove (17)
- ReHand (18)
- ReIn-Hand system (19)
- RUPERT (20)
- SaeboMAS (21)
- SCRIPT Active Orthosis (22)
- SEM Glove (23)
- SNU Exo-Glove (24)
- Tenoexo hand exoskeleton (25)
- TENS Stimulator (26)
- TIGER (27)
- X-Glove (28)
- Other (please specify) (29) __________________________________________________

***Display This Question: If Q12 = No***

##### Q15: Do you feel you have enough access to assistive devices for hands and wrists?

- Definitely yes (1)
- Probably yes (2)
- May or may not (3)
- Probably not (4)
- Definitely not (5)

***Display This Question***:

***If Q8 = Yes***
***Or Q9 = Yes***
***Or Q12 = Yes***

##### Q16: In your opinion, which factors are most important when assessing an assistive device?

**Table:**

	Not at all important (1)	Slightly important (2)	Moderately important (3)	Very important (4)	Extremely important (5)
Cost of device (1)	○	○	○	○	○
Ease of use (2)	○	○	○	○	○
Mechanical power (3)	○	○	○	○	○
Portability (4)	○	○	○	○	○
Safety (5)	○	○	○	○	○
User satisfaction (6)	○	○	○	○	○
Wearability and comfort (7)	○	○	○	○	○
Weight (8)	○	○	○	○	○

#### OUTCOME MEASURES

##### Q18: Do you have experience using outcome measures for upper limb assessment?

- Yes (1)
- Some experience (2)
- No (3)

***Display This Question: If Q18!= No***

##### Q19: Which outcome measure(s) do you have experience with?

- Action Research Arm Test (ARAT) (1)
- Ashworth scale (2)
- Box and Blocks Test (BBT) (3)
- Disabilities of the Arm Shoulder and Hand Questionnaire (DASH) (4)
- Force Control tests (5)
- Fugl-Meyer Assessment (FMA) (6)
- Graded Redefined Assessment of Strength, Sensibility and Prehension (GRASSP) (7)
- Jebsen-Taylor Hand Function tests (JTHFT) (8)
- Motor Activity Log (MAL) (9)
- Motor Assessment Scale (MAS) (10)
- Nine Hole Peg Test (9HPT) (11)
- Pain (self-reported) (12)
- Purdue Pegboard test (PPT) (13)
- Range of Motion tests (14)
- Southampton Hand Assessment Procedure (SHAP) (15)
- Wolf Motor Function Test (WMFT) (16)
- Other (please specify) (17) _______________________________________________

***Display This Question: If Q18!= No***

##### Q20: Which outcome measure(s) do you recommend using for hand and/or wrist assessment?

- Action Research Arm Test (ARAT) (1)
- Ashworth scale (2)
- Box and Blocks Test (BBT) (3)
- Disabilities of the Arm Shoulder and Hand Questionnaire (DASH) (4)
- Force Control tests (5)
- Fugl-Meyer Assessment (FMA) (6)
- Graded Redefined Assessment of Strength, Sensibility and Prehension (GRASSP) (7)
- Jebsen-Taylor Hand Function tests (JTHFT) (8)
- Motor Activity Log (MAL) (9)
- Motor Assessment Scale (MAS) (10)
- Nine Hole Peg Test (9HPT) (11)
- Pain (self-reported) (12)
- Purdue Pegboard test (PPT) (13)
- Range of Motion tests (14)
- Southampton Hand Assessment Procedure (SHAP) (15)
- Wolf Motor Function Test (WMFT) (16)
- None (17)
- Other (please specify) (18)

##### Q21: What do you feel are the main limitations to assessing outcomes for patients requiring hand and wrist assistive devices?

- Time with patients (1)
- Lack of skills or training (2)
- Lack of equipment (3)
- Other (please explain) (4) __________________________________________________

##### Q22: In your opinion, are outcome measurement tools useful for patients requiring assistive devices?

- Yes (1)
- Unsure (2)
- No (3)

##### Q23: In your opinion, could outcome measurement tools be improved for patients requiring assistive devices?

- Yes (1)
- Unsure (2)
- No (3)

***Display This Question: If Q23 = Yes***
